# Supplementary material for: BK channel properties correlate with neurobehavioral severity in three KCNMA1-linked channelopathy mouse models
Source: eLife. 2022 Jul 12;11:e77953. doi: 10.7554/eLife.77953 (PMC9275823; doi:10.7554/eLife.77953)
Supplement: Source code 1. — Python code to calculate wheel speed and the number and duration of activity gaps. [file elife-77953-code1.zip › 16-02-2022-RA-eLife-77953/Wheel Activity Python Script 2021.pdf]

```
'''
Meredith Lab
Created by Cooper Roache

Must have openpyxl installed through pip

This script will read in the exported time series Excel (.xlsx) spreadsheet created by Clocklab
Reads in 12-hour dark phase (assuming start time is set to light cycle start) and total daily wheel rotation counts
Only for period=24hr in 12-hour light/dark cycle

Arguments after command to run script:
#1 - .xlsx filename/location
#2 - Run duration rpm threshold > 0rpm
#3 - Allowed consecutive 0rpm bins/consecutive bins falling below rpm threshold (gap size)

Will calculate:
- total rotation count, average 12-hour dark phase rotation count
- average & maximum speed
- average, maximum and average daily maximum run duration, average rotation count per run event and run event count
- average, maximum and average daily maximum activity gap duration, activity gap event count

Note:
- All calculated values are over 12-hour dark phase except total rotation count (i.e. grand total)
- A run duration event is defined as consecutive bin values over a user defined threshold (rpm)
without falling below said threshold for a user defined number of bins (gap size)
- An activity gap duration event is defined as consecutive bin values of 0rpm
with a user defined number of consecutive 0rpm values allowed (gap size)
'''

#!usr/bin/env python3

import sys
import os
import openpyxl
import math

#Enter the .xlsx filename/location (arg 1) and run duration rpm threshold > 0rpm (arg 2)
usr_input = sys.argv

if len(usr_input) == 4:
	filename = usr_input[1]

	if not ".xlsx" in str(filename):
		print("Please enter a valid .xlsx file")

		sys.exit(1)
```

```
	try:
		rpm_thres = float(usr_input[2])
	except:
		print("Please enter a valid float for run duration rpm threshold (arg 2)")

		sys.exit(1)

	try:
		gap_size = int(usr_input[3])
	except:
		print("Please enter a valid integer for the allowed activity gap duration (arg 3)")

		sys.exit(1)
else:
	print("Please enter only a .xlsx file location (arg 1), run duration event (float) rpm threshold (arg 2) and allowed activity gap (int) duration (arg 3)")

	sys.exit(1)

if rpm_thres <= 0:
	print("Please enter a run duration rpm threshold greater than 0 (arg 2)")

	sys.exit(1)
elif gap_size < 0 or isinstance(gap_size, int) == False:
	print("Please enter an allowed activity gap greater than or equal to 0 (arg 3)")

	sys.exit(1)

#Try to open excel file
if os.path.exists(filename) == True:
	wb = openpyxl.load_workbook(filename, data_only = True)
else:
	print("No such .xlsx file or directory: '" + filename + "'")

	sys.exit(1)

#Use active sheet in file
sheet = wb.active

#Creating total list of all 24-hour rotation counts
total_list = []

for row in range(4, 1000000):
	hour = sheet["B" + str(row)].value
	value = sheet["D" + str(row)].value

	#Checking if wheel count value exists for each bin and appending list
```

```
        if isinstance(hour, int) == False:
                break
        else:
                if isinstance(value, int):
                        total_list.append(value)

#Defining light and dark phase start times
light_start = sheet["B4"].value
dark_start = light_start + 12

#Calculating blocksize and number of bins per dark phase
blocksize = sheet["C5"].value - sheet["C4"].value
bins_per_dark_phase = (60 / blocksize) * 12

#Creating list from all bin values in 12-hour dark phase
dark_list = []

for row in range(4, 1000000):
        hour = sheet["B" + str(row)].value
        next_hour = sheet["B" + str(row + 1)].value
        value = sheet["D" + str(row)].value
        next_dark_phase = sheet["B" + str(row + int(bins_per_dark_phase))].value

        #Checking if the wheel count value exists for each bin and appending list
        if isinstance(value, int):
                if hour >= dark_start or hour < light_start:
                        dark_list.append(value)

        #Checking if upcoming dark phase is a full 12 hours
        if isinstance(next_hour, int):
                if (row - 3) % bins_per_dark_phase == 0 and next_hour >= dark_start:
                        if isinstance(next_dark_phase, int) == False:
                                break
        else:
                break

#Closing workbook
wb.close()

##########################################################################################
##########################

#Number of total 12-hour dark phase days analyzed
days_analyzed = int(len(dark_list) / bins_per_dark_phase)

#Iterate through all bin values in 12-hour dark phase list
dark_list_total = 0
```

```
avg_count = 0
avg_count_list = []

max_speed = 0
max_speed_list = []

duration_count = 0
avg_duration_count = 0
max_duration = 0
duration_count_list = []
avg_duration_list = []
max_duration_list = []

gap_duration_count = 0
max_gap_duration = 0
gap_duration_list = []
max_gap_duration_list = []

gap_sized_temp_list = []
zero_first = False
gap_duration_adj = gap_size * blocksize

for num in range(0, len(dark_list)):
        value = dark_list[num]
        temp_list_sum = 0

        #Checking if the first value of the 12-hour dark phase list is 0
        if value == 0 and num == 0:
                zero_first = True

        #Maintaining temporary list (length of gap size + 1) of last values
        if gap_size > 0:
                if len(gap_sized_temp_list) <= gap_size:
                        gap_sized_temp_list.append(value)
                else:
                        gap_sized_temp_list.pop(0)

                        gap_sized_temp_list.append(value)

        #Sumation of a bin values in 12-hour dark phase list
        dark_list_total += value

        #Checking if current value is the max speed
        if value > max_speed:
                max_speed = value

        #Checking if any of the temp values in the gap sized temp list are over rpm threshold
        if gap_size > 0:
```

```
            for temp in gap_sized_temp_list:
                temp_list_sum += temp

                if temp >= rpm_thres:
                    over_rpm_thres = True

                    break
                else:
                    over_rpm_thres = False
        #If gap size = 0
        else:
            if value >= rpm_thres:
                over_rpm_thres = True
            else:
                over_rpm_thres = False

        #Checking if any of the last 5 values are over the rpm threshold (from temp list)
        if over_rpm_thres == True:
            #Adding to run event count and average duration count
            duration_count += value
            avg_duration_count += blocksize
        else:
            #Appending to duration event list and reset duration count
            if avg_duration_count > 0:
                if gap_size > 0:
                    #Subtracting values that counted past run event stop due to gap size
                    duration_count -= temp_list_sum - (gap_sized_temp_list[gap_size])
                    avg_duration_count -= gap_duration_adj

                avg_duration_list.append(avg_duration_count)
                duration_count_list.append(duration_count)

                #Checking if current duration is the max duration
                if avg_duration_count > max_duration:
                    max_duration = avg_duration_count

                avg_duration_count = 0
                duration_count = 0

        #Adding to gap event count
        if (len(gap_sized_temp_list) > 0 and temp_list_sum == 0) or (len(gap_sized_temp_list) == 0 and
value == 0):
            gap_duration_count += blocksize
        else:
            #Appending to gap duration event list and reset gap duration count
            if gap_duration_count > 0:
                #Adding values that did not count in prior iterations due to gap size
                gap_duration_count += gap_duration_adj
```

```
                        gap_duration_list.append(gap_duration_count)

                        #Checking if current gap duration is the max gap duration
                        if gap_duration_count > max_gap_duration:
                                max_gap_duration = gap_duration_count

                        gap_duration_count = 0

        #Appending average max per day lists based on dividing loop number by bins per dark phase
        if (num + 1) % bins_per_dark_phase == 0:
                max_speed_list.append(max_speed)
                max_duration_list.append(max_duration)
                max_gap_duration_list.append(max_gap_duration)

                max_speed = 0
                max_duration = 0
                max_gap_duration = 0

#Either popping off or adjusting first item in gap event list if there is a 0 as the first value
if zero_first == True and (gap_duration_list[0] - gap_duration_adj) <= gap_size:
        gap_duration_list.pop(0)
elif zero_first == True and (gap_duration_list[0] - gap_duration_adj) > gap_size:
        gap_duration_list[0] -= gap_duration_adj

#####################################################################################
##########################

#Calculating grand total rotation count from all 24-hour rotation counts
total_count = 0

for total in total_list:
        total_count += total

#Calculating average dark phase rotation count
if days_analyzed > 0:
        avg_count = dark_list_total / days_analyzed
else:
        print("Unable to calculate average rotation count: 0 12-hour dark phases analyzed\n")

#Calculating average speed
avg_speed = 0

if len(dark_list) > 0:
        avg_speed = dark_list_total / len(dark_list)
else:
        print("Total count list empty\n")
```

```
#Calculating max speed and average max speed per day
overall_max_speed = 0
max_speed_list_sum = 0
avg_max_speed = 0

if len(max_speed_list) > 0:
	for speed in max_speed_list:
		max_speed_list_sum += speed

		if speed > overall_max_speed:
			overall_max_speed = speed

	avg_max_speed = max_speed_list_sum / len(max_speed_list)
else:
	print("Max speed list empty\n")

#Calculating average run duration from run duration event list
duration_list_sum = 0
avg_duration = 0

if len(avg_duration_list) > 0:
	for duration_event in avg_duration_list:
		duration_list_sum += duration_event

	avg_duration = duration_list_sum / len(avg_duration_list)
else:
	print("Run duration event list empty\n")

#Calculating max run duration overall and average max run duration per day
max_duration_list_sum = 0
overall_max_duration = 0
avg_max_duration = 0

if len(max_duration_list) > 0:
	for duration_event in max_duration_list:
		max_duration_list_sum += duration_event

		if duration_event > overall_max_duration:
			overall_max_duration = duration_event

	avg_max_duration = max_duration_list_sum / len(max_duration_list)
else:
	print("Max run duration event list empty\n")

#Calculating average run duration rotation count
duration_count_list_sum = 0
avg_duration_count = 0
```

```
if len(duration_count_list) > 0:
	for duration_event in duration_count_list:
		duration_count_list_sum += duration_event

	avg_duration_count = duration_count_list_sum / len(duration_count_list)
else:
	print("Run duration count list empty\n")

#Calculating average gap duration from gap duration event list
gap_duration_list_sum = 0
avg_gap_duration = 0

if len(gap_duration_list) > 0:
	for gap_event in gap_duration_list:
		gap_duration_list_sum += gap_event

	avg_gap_duration = gap_duration_list_sum / len(gap_duration_list)
else:
	print("Gap duration event list empty\n")

#Calculating max gap duration overall and average max gap duration per day
max_gap_duration_list_sum = 0
overall_max_gap_duration = 0
avg_max_gap_duration = 0

if len(max_gap_duration_list) > 0:
	for gap_event in max_gap_duration_list:
		max_gap_duration_list_sum += gap_event

		if gap_event > overall_max_gap_duration:
			overall_max_gap_duration = gap_event

	avg_max_gap_duration = max_gap_duration_list_sum / len(max_gap_duration_list)
else:
	print("Max gap duration event list empty\n")

#Calculating average run and activity gap events per day
avg_run_events = 0
avg_gap_events = 0

if days_analyzed > 0:
	avg_run_events = len(avg_duration_list) / days_analyzed
	avg_gap_events = len(gap_duration_list) / days_analyzed
else:
	print("Unable to calculate average events: 0 days analyzed\n")

###########################################################################################
###########################
```

```
#Unit conversions
conv_factor = 0.1190625 * math.pi

total_count_m = total_count * conv_factor
avg_count_m = avg_count * conv_factor

avg_speed_m_per_s = avg_speed * conv_factor / 60
max_speed_m_per_s = overall_max_speed * conv_factor / 60
avg_max_speed_m_per_s= avg_max_speed * conv_factor / 60

avg_duration_sec = avg_duration * 60
max_duration_sec = overall_max_duration * 60
avg_max_duration_sec = avg_max_duration * 60

avg_gap_duration_sec = avg_gap_duration * 60
max_gap_duration_sec = overall_max_gap_duration * 60
avg_max_gap_duration_sec = avg_max_gap_duration * 60

#Converting start time into 24-hour clock time
value_minute_start = sheet["C4"].value
minute_start = str(value_minute_start)

if value_minute_start >= 0 and value_minute_start < 10:
	minute_start = "0" + minute_start

light_start_clock = str(light_start) + ":" + minute_start

#Print all parameter descriptions in order
print("12-hour light phase start time: " + str(light_start_clock))
print("Blocksize: " + str(blocksize) + "min")
print("Run event RPM threshold: " + str(rpm_thres) + "rpm")
print("Allowed activity gap/count under RPM threshold: " + str(gap_size * blocksize) + "min")
print("Number of 12-hour dark phases analyzed: " + str(days_analyzed))
print("\n")

print("Results appear in the order described below:")
print("1. Total 24-hour rotation count")
print("2. Average 12-hour rotation count")

print("3. Average speed")
print("4. Maximum speed")
print("5. Average maximum speed per day")

print("6. Average run duration")
print("7. Maximum run duration over")
print("8. Average maximum run duration per day")
print("9. Average rotation count per run")
```

```
print("10. Average number of run events per day")

print("11. Average activity gap duration")
print("12. Maximum activity gap duration")
print("13. Average maximum activity gap duration per day")
print("14. Average number of activity gap events per day")
print("\n")

#Print all results in order
print("All results in minutes (min) and rotations per minute (rpm):")

print(total_count)
print(round(avg_count, 2))

print(round(avg_speed, 2))
print(overall_max_speed)
print(round(avg_max_speed, 2))

print(round(avg_duration, 2))
print(overall_max_duration)
print(round(avg_max_duration, 2))
print(round(avg_duration_count, 2))
print(round(avg_run_events, 2))

print(round(avg_gap_duration, 2))
print(overall_max_gap_duration)
print(round(avg_max_gap_duration, 2))
print(round(avg_gap_events, ))
print("\n")

#Print all unit conversions
print("All results in meters (m), seconds (s) and meters per second (m/s):")

print(round(total_count_m, 2))
print(round(avg_count_m, 2))

print(round(avg_speed_m_per_s, 2))
print(round(max_speed_m_per_s, 2))
print(round(avg_max_speed_m_per_s, 2))

print(round(avg_duration_sec, 2))
print(max_duration_sec)
print(round(avg_max_duration_sec, 2))
print(round(avg_duration_count, 2))
print(round(avg_run_events, 2))

print(round(avg_gap_duration_sec, 2))
print(max_gap_duration_sec)
```

```
print(round(avg_max_gap_duration_sec, 2))
print(round(avg_gap_events, 2))

sys.exit(0)
```
