# Supplementary material for: BK channel properties correlate with neurobehavioral severity in three KCNMA1-linked channelopathy mouse models
Source: eLife. 2022 Jul 12;11:e77953. doi: 10.7554/eLife.77953 (PMC9275823; doi:10.7554/eLife.77953)
Supplement: Transparent reporting form [file elife-77953-transrepform1.pdf]

eLIFE
1st Floor
24 Hills Road
Cambridge CB2 1JP, UK
P 01223 855340
W elifesciences.org
T @elife

eLife's transparent reporting form

We encourage authors to provide detailed information *within their submission* to facilitate the interpretation and replication of experiments. Authors can upload supporting documentation to indicate the use of appropriate reporting guidelines for health-related research (see EQUATOR Network), life science research (see the BioSharing Information Resource), or the ARRIVE guidelines for reporting work involving animal research. Where applicable, authors should refer to any relevant reporting standards documents in this form.

If you have any questions, please consult our Journal Policies and/or contact us: editorial@elifesciences.org.

Sample-size estimation

- You should state whether an appropriate sample size was computed when the study was being designed
- You should state the statistical method of sample size computation and any required assumptions
- If no explicit power analysis was used, you should describe how you decided what sample (replicate) size (number) to use

Please outline where this information can be found within the submission (e.g., sections or figure legends), or explain why this information doesn't apply to your submission:

Sample size estimates were based on prior studies in our lab and within the literature. The sample size for each group is found within the figure legends:
Figure 1: n=9-12
Figure 2: N999S group: n=14-16; D434G group: n=9-12; H444Q group: n=6-7.
Figure 3: N999S group: n=16-23; D434G group: n=19-27; H444Q group: n=7-11.
Figure 4: N999S group: n=13-18; D434G group: n=7-11; H444Q group: n=4-7; *Kcnma1*$^{-/-}$group: n= 9-11.
Figure 5: Since prior sample size estimates were not available for the novel restraint stress-induced immobility assay, sample size estimates were determined from N999S HET (n=4) and WT (n=4) in a pilot study using power analysis with G*power 3.1. The effect size of d=2.29 at 0.8 power level with 0.05 α error probability required n=5 (Methods). Actual group sizes: N999S group: n=7-11; D434G group: n=7-18; H444Q group: n=3-11; Kcnma1$^{-/-}$ group: n= 8-11.

Replicates

- You should report how often each experiment was performed
- You should include a definition of biological versus technical replication
- The data obtained should be provided and sufficient information should be provided to indicate the number of independent biological and/or technical replicates
- If you encountered any outliers, you should describe how these were handled
- Criteria for exclusion/inclusion of data should be clearly stated
- High-throughput sequence data should be uploaded before submission, with a private link for reviewers provided (these are available from both GEO and ArrayExpress)

eLife Sciences Publications, Ltd is a limited liability non-profit non-stock corporation incorporated in the State of Delaware, USA, with company number 5030732, and is registered in the UK with company number FC030576 and branch number BR015634 at the address 1st Floor, 24 Hills Road, Cambridge CB2 1JP | August 2014

Please outline where this information can be found within the submission (e.g., sections or figure legends), or explain why this information doesn't apply to your submission:

The following information is found in the methods.
Figure 1 and 2: Three current responses were averaged to obtain BK current parameters (technical replicate). Individual patches were biological replicates.
Figure 3: A single current injection protocol was run per neuron (no technical replicate). Individual neurons were biological replicates.
Figures 4-5: A single EEG or behavioral assay was run per animal (no technical replicate). Individual animals were biological replicates.
In all datasets, outliers were included. Data exclusion for technical errors is stated in methods.

Statistical reporting

- Statistical analysis methods should be described and justified
- Raw data should be presented in figures whenever informative to do so (typically when N per group is less than 10)
- For each experiment, you should identify the statistical tests used, exact values of N, definitions of center, methods of multiple test correction, and dispersion and precision measures (e.g., mean, median, SD, SEM, confidence intervals; and, for the major substantive results, a measure of effect size (e.g., Pearson's r, Cohen's d)
- Report exact p-values wherever possible alongside the summary statistics and 95% confidence intervals. These should be reported for all key questions and not only when the p-value is less than 0.05.

Please outline where this information can be found within the submission (e.g., sections or figure legends), or explain why this information doesn't apply to your submission:

Rationale for each statistical test based on data structure and multiple test corrections are described in the Methods section. Statistical test, N, mean or median, SEM, and exact p-values are described in the Results section and each figure legend. Detailed statistical parameters (F-statistics, etc) beyond those stated in the figure legends are found the Excel file 'Supplemental Statistical Parameters'

(For large datasets, or papers with a very large number of statistical tests, you may upload a single table file with tests, Ns, etc., with reference to sections in the manuscript.)

Group allocation

- Indicate how samples were allocated into experimental groups (in the case of clinical studies, please specify allocation to treatment method); if randomization was used, please also state if restricted randomization was applied
- Indicate if masking was used during group allocation, data collection and/or data analysis

Please outline where this information can be found within the submission (e.g., sections or figure legends), or explain why this information doesn't apply to your submission:

The following information is found in the Methods.

Heterologous cell recordings:
Figure 1: No blinding was employed. BK^{WT}, BK^{N999S}, BK^{D434G}, and BK^{H444Q} constructs were allocated into the same cell transfections on the same days.

Mice were allocated into experimental groups as sex-matched WT and transgenic littermates where possible. Blinded data collection is stated for all animal experiments as follows:
Figure 2-5: Experimenters were blinded to genotype at data collection and parameter analysis. In Figure 5C, the experimenter was blinded to both genotype and d-amphetamine dose. A separate blinded experiment conducted an independent data analysis.

Additional data files ("source data")

- We encourage you to upload relevant additional data files, such as numerical data that are represented as a graph in a figure, or as a summary table

- Where provided, these should be in the most useful format, and they can be uploaded as "Source data" files linked to a main figure or table
- Include model definition files including the full list of parameters used
- Include code used for data analysis (e.g., R, MatLab)
- Avoid stating that data files are "available upon request"

Please indicate the figures or tables for which source data files have been provided:

The CRISPR guide and *KCNMA1* target sequences used to generate transgenic mice are included as Figure 1-figure supplement 1. A summary 'Supplemental Document for Reagents' is added to the beginning of the methods. The source data for all experiments is provided as a zipped set of Prism files. The python code is provided for the wheel running analysis script.

eLife Sciences Publications, Ltd is a limited liability non-profit non-stock corporation incorporated in the State of Delaware, USA, with company number 5030732, and is registered in the UK with company number FC030576 and branch number BR015634 at the address 1st Floor, 24 Hills Road, Cambridge CB2 1JP | August 2014
